# Puerarin‐Loaded Electrospun Patches with Anti‐Inflammatory and Pro‐Collagen Synthesis Properties for Pelvic Floor Reconstruction

**DOI:** 10.1002/advs.202308590

**Published:** 2024-03-21

**Authors:** Di Zhang, Dong Xu, Xiaobo Huang, Yingqi Wei, Fuxin Tang, Xiusen Qin, Weiwen Liang, Zhongping Liang, Lin Jin, Hui Wang, Huaiming Wang

**Affiliations:** ^1^ Department of General Surgery (Colorectal Surgery) Guangdong Provincial Key Laboratory of Colorectal and Pelvic Floor Diseases Guangdong Institute of Gastroenterology Biomedical Innovation Center The Sixth Affiliated Hospital Sun Yat‐sen University Guangzhou 510655 China; ^2^ Department of Ophthalmology Biomedical Innovation Center The Sixth Affiliated Hospital Sun Yat‐sen University Guangzhou 510655 China; ^3^ Translational Medical Center The First Affiliated Hospital of Zhengzhou University Zhengzhou 450052 China; ^4^ The Sixth Affiliated Hospital of Guangzhou Medical University Qingyuan People's Hospital China; ^5^ International Joint Research Laboratory for Biomedical Nanomaterials of Henan Zhoukou Normal University Zhoukou 466001 China

**Keywords:** anti‐inflammatory, drug delivery, electrospun patch, pelvic organ prolapse, pro‐collagen synthesis

## Abstract

Pelvic organ prolapse (POP) is one of the most common pelvic floor dysfunction disorders worldwide. The weakening of pelvic connective tissues initiated by excessive collagen degradation is a leading cause of POP. However, the patches currently used in the clinic trigger an unfavorable inflammatory response, which often leads to implantation failure and the inability to simultaneously reverse progressive collagen degradation. Therefore, to overcome the present challenges, a new strategy is applied by introducing puerarin (Pue) into poly(l‐lactic acid) (PLLA) using electrospinning technology. PLLA improves the mechanical properties of the patch, while Pue offers intrinsic anti‐inflammatory and pro‐collagen synthesis effects. The results show that Pue is released from PLLA@Pue in a sustained manner for more than 20 days, with a total release rate exceeding 80%. The PLLA@Pue electrospun patches also show good biocompatibility and low cytotoxicity. The excellent anti‐inflammatory and pro‐collagen synthesis properties of the PLLA@Pue patch are demonstrated both in vitro in H_2_O_2_‐stimulated mouse fibroblasts and in vivo in rat abdominal wall muscle defects. Therefore, it is believed that this multifunctional electrospun patch integrating anti‐inflammatory and pro‐collagen synthesis properties can overcome the limitations of traditional patches and has great prospects for efficient pelvic floor reconstruction.

## Graphical abstract

To overcome the defects of traditional meshes, which are easy to cause inflammatory responses and cannot reverse collagen degradation, a drug‐loaded electrospun patch is developed for the treatment of pelvic organ prolapse. Poly(l‐lactic acid) improves the mechanical properties of the patch, while puerarin can be released in a sustained manner and offers intrinsic anti‐inflammatory and pro‐collagen synthesis effects to reduce complications and recurrence.

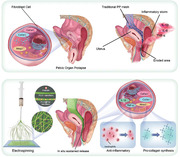

## 1 Introduction

Pelvic organ prolapse (POP) is a common urogynecological disorder that affects ≈25% of women across all age groups.^[^ ^1^ ^]^ The main symptoms usually involve multiple organs, including urinary incontinence, vaginal bulge, fecal incontinence, and sexual dysfunction. With the aging trend of the global population, the prevalence of POP continues to rise.^[^ ^2^ ^]^ Up to 19% of these women will undergo at least one surgical intervention for POP during their lifetime at a cost of at least one billion dollars in medical expenses annually.^[^ ^3^ ^]^ POP has increasingly become a psychological and economic burden worldwide. There is considerable evidence that weakened pelvic connective tissues, which mainly consist of fibroblasts and their products (collagen), play an essential role in the pathophysiology of POP.^[^ ^4^ ^]^ Decreased collagen synthesis and increased collagen degradation are thought to be factors that contribute to POP.^[^ ^5^ ^]^ Traditional surgical procedures using patients’ native tissue repair to strengthen the pelvic supportive structures have gradually been replaced by procedures using mesh for reconstruction due to the high rate of recurrence.^[^ ^6^ ^]^

Nondegradable, lightweight synthesized meshes with mechanical durability (e.g., polypropylene (PP) meshes) have been widely used in pelvic reconstruction surgery for POP.^[^ ^7^ ^]^ However, these meshes bear a 29–50% risk of serious postsurgical complications, such as exposure, retraction, and erosion.^[^ ^8^ ^]^ These complications prompted the Food and Drug Administration (FDA) to issue statement warnings for the clinical application of these meshes in 2007 and 2011, leading to market withdrawals and bans on commercial transvaginal mesh products in several countries.^[^ ^9^ ^]^ This phenomenon arises from the poor biocompatibility between pelvic tissue and the PP mesh, resulting in an undesirable inflammatory response.^[^ ^10^ ^]^ Moreover, due to the lack of microstructures suitable for cell migration and growth, the implanted nondegradable PP mesh cannot reverse the nature of progressive collagen degradation in the pelvic connective tissues of POP patients.^[^ ^5^, ^11^ ^]^ Therefore, determining how to design patches that integrate anti‐inflammatory and pro‐collagen synthesis properties is the key to improving the effect of pelvic reconstruction for POP treatment.

Electrospinning is a unique technique that is used to prepare micro/nanosized fibers with a 3D structure.^[^ ^12^ ^]^ Because of their good biocompatibility, superior flexibility and tensile performance, and a structure that is similar to native extracellular matrix (ECM), electrospun patches have been gradually regarded as attractive candidates for pelvic reconstruction.^[^ ^13^ ^]^ Among them, biodegradable poly(l‐lactic acid) (PLLA) electrospun patches have been reported by several research groups for repairing pelvic floor fascia due to their standardized production, controllable degradation rate and mechanical strength that is close to that of healthy pelvic soft tissues.^[^ ^4^, ^14^ ^]^

In addition to biomaterials, drugs or bioactive factors are additional key factors in pelvic floor tissue engineering, as they can endow biomaterials with the ability to regulate immune‐mediated inflammation in the pelvic microenvironment and to continuously promote collagen synthesis in fibroblasts.^[^ ^4^, ^15^ ^]^ With a large surface area‐to‐volume ratio and high porosity, electrospun patches are an ideal drug delivery carrier. Drugs can be easily incorporated into electrospun fibers and released over a long time.^[^ ^16^ ^]^ For example, Mangır and colleagues prepared poly(lactic acid) (PLA)‐based ascorbic acid‐releasing electrospun nanofibers and found that the ascorbic acid‐loaded nanofibers could significantly increase collagen deposition and therefore have the potential to enhance natural tissue repair in POP applications.^[^ ^14a^ ^]^ Some electrospun patches loaded with bioactive factors, such as gelatin^[^ ^17^ ^]^ or fibroblast growth factor (bFGF),^[^ ^4^ ^]^ have good biocompatibility and can reduce the inflammatory response caused by patch implantation. However, at present, electrospun patches modified by drugs or loaded with bioactive factors have only anti‐inflammatory or pro‐collagen synthesis functions, but not both. Therefore, the key to the successful application of electrospun nanofibers in the field of pelvic reconstruction is simultaneously producing anti‐inflammatory effects and promoting collagen synthesis with a single electrospun patch. Puerarin (Pue) is a natural isoflavone glycoside extracted from *Pueraria lobata* OHWI (Wild) roots, a traditional Chinese herbal medicine.^[^ ^18^ ^]^ Several studies have shown that Pue is useful for treating acute kidney injury, osteoporosis, osteoarthritis, and other inflammation‐related diseases by effectively inhibiting the release of proinflammatory cytokines such as IL‐1β, IL‐6, and TNF‐α.^[^ ^19^ ^]^ Moreover, Li and colleagues found that Pue could promote the synthesis of collagen in fibroblasts in POP patients by regulating the expression of matrix metalloproteinases (Mmps).^[^ ^20^ ^]^

**Figure 1:**
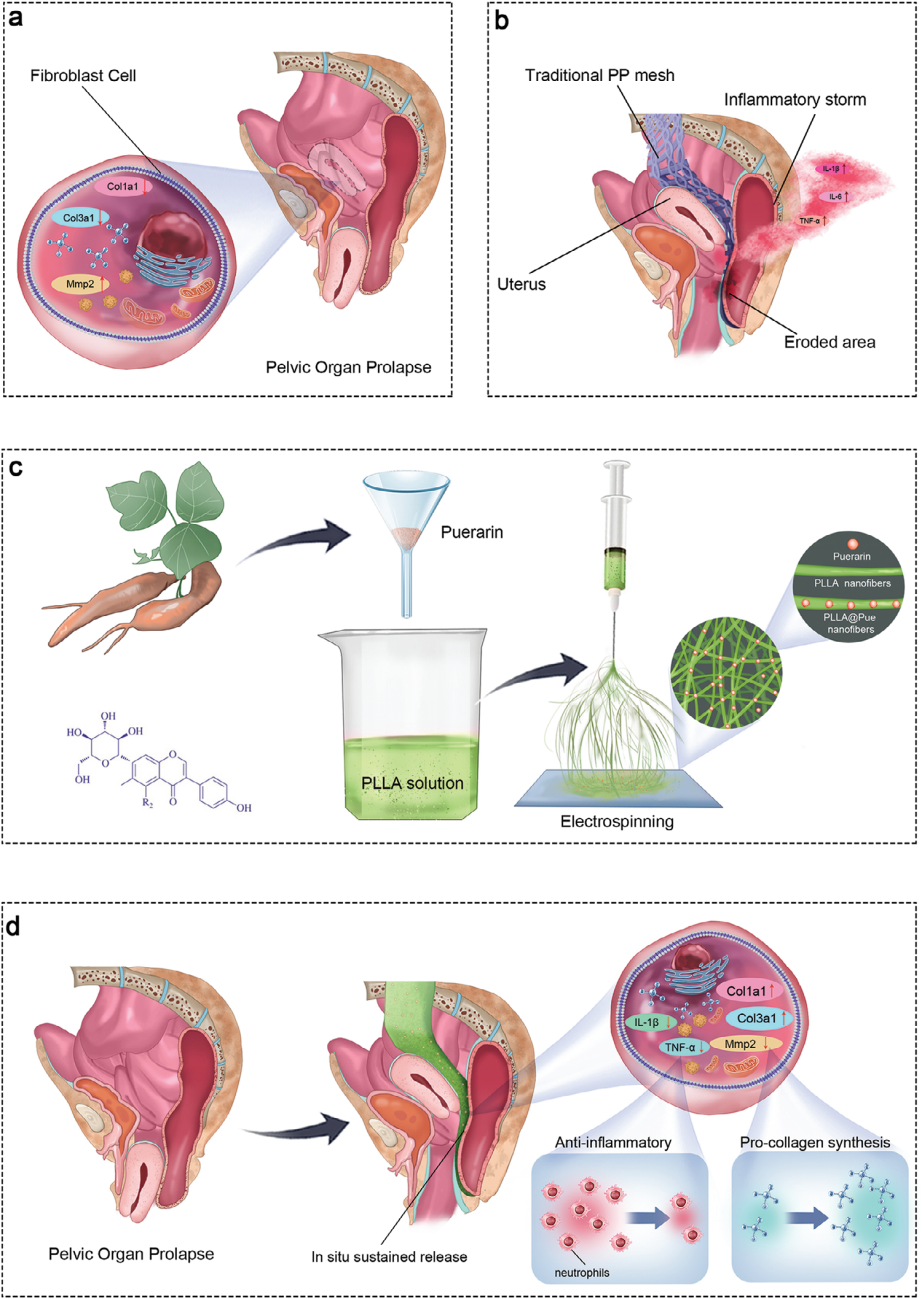
Schematic illustration of PLLA@Pue for the treatment of POP. a) The pathogenesis of POP in molecular. b) Traditional PP meshes often cause serious complications such as mesh erosion due to the undesirable inflammatory response. c) Preparation of PLLA@Pue and its internal microstructure. d) The PLLA@Pue promotes pelvic floor repair through the combined anti‐inflammatory and pro‐collagen synthesis effects.

Herein, inspired by the undesirable inflammatory response after biomaterial implantation and progressive collagen degradation, a new class of electrospun patch carrying Pue, PLLA@Pue, was designed to reduce the local inflammatory response and promote the repair of pelvic floor ligaments (**Figure** **1**). In the prepared PLLA@Pue electrospun patch, the PLLA component provides good biocompatibility and mechanical properties. Moreover, Pue can be continuously released from the patch, which not only inhibits the inflammatory response by reducing the expression of proinflammatory cytokines but also promotes pelvic tissue repair by regulating collagen synthesis. The therapeutic effect of this patch was investigated using mouse fibroblasts under a hydrogen peroxide (H_2_O_2_)‐stimulated inflammatory environment in vitro. A rat abdominal wall muscle defect model was established and used to evaluate its anti‐inflammatory and pro‐collagen synthesis abilities as well as safety in vivo. We hope that our PLLA@Pue patch will become a promising material in pelvic reconstruction for POP treatment.

## 2 Results and Discussion

### 2.1 Characterization of the Electrospun Patch

**Figure 2:**
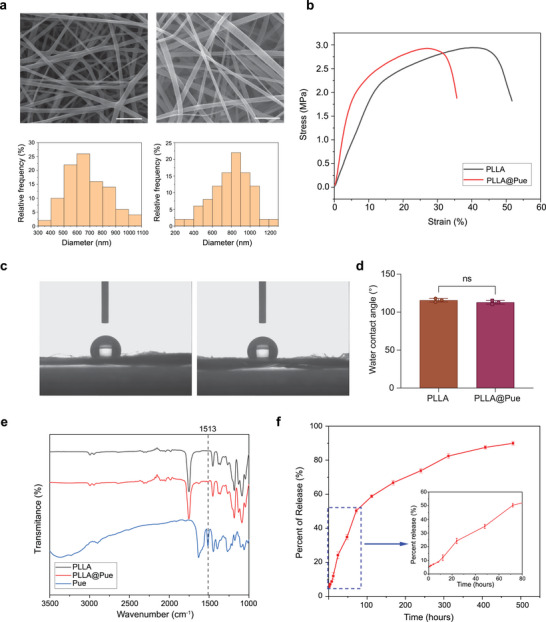
Characteristics of the electrospun patches. a) SEM images and the diameter frequency distribution map of PLLA and PLLA@Pue. b) The stress‐strain test of PLLA and PLLA@Pue. c and d) The water contact angle of PLLA and PLLA@Pue. e) FT‐IR spectra of Pue, PLLA, and PLLA@Pue. f) The releasing curve of Pue from PLLA@Pue. Scale bars: 5 µm.

Electrospun patches have been widely used in tissue engineering because they can simulate the microstructure of the native ECM.^[^ ^21^ ^]^ **Figure** **2**a shows the surface morphologies of the PLLA and PLLA@Pue electrospun patches. The scanning electron microscopy (SEM) images showed that the two groups of electrospun fibers had good uniformity and an irregular arrangement. The fiber surface was smooth and not porous. The PLLA fiber diameter was 685 ± 170 nm, and that of PLLA@Pue was 795 ± 210 nm. Pue loading slightly increased the fiber diameter but had no influence on the surface morphology or structure of the PLLA electrospun fibers.

Mechanical properties play a vital role in patches used for pelvic floor reconstruction because decreases in mechanical properties are often associated with a high recurrence rate after POP surgery.^[^ ^22^ ^]^ To explore the mechanical properties of the PLLA and PLLA@Pue electrospun patches, stress‒strain tests were carried out. As shown in Figure 2b, the stretching curves of PLLA and PLLA@Pue were similar. The tensile strengths of PLLA and PLLA@Pue were 2.94 ± 0.32 and 2.93 ± 0.31 MPa, respectively. These results indicated that the addition of Pue did not affect the mechanical properties of the PLLA electrospun patches. The ultimate tensile strength of PLLA@Pue was similar to that of other reported electrospun patches used for POP,^[^ ^4^, ^8^ ^]^ and significantly higher than that of patches made of hydrogel.^[^ ^10a^ ^]^ Meanwhile, according to the stress‐strain curves, we calculated that Young's modulus of PLLA and PLLA@Pue were 10.67 ± 0.73 and 23.41 ± 0.81 Mpa, respectively. While the Young's modulus of vaginal tissue from women with POP was reported 1.94–3.43 Mpa,^[^ ^23^ ^]^ so vaginal tissue is prone to deformation when abdominal pressure increases. Therefore, PLLA@Pue is qualified to meet the demand of the high‐tension environment for pelvic floor reconstruction and provide good support for tissue regeneration. As shown in Figure 2c,d, the water contact angles (WCAs) of the PLLA and PLLA@Pue electrospun patches were 115.7 ± 2.3° and 113.0 ± 2.4°, respectively. There was no significant difference between them, indicating that the addition of Pue did not change the wettability of the nanofiber scaffold.

To determine the effect Pue had on the structure of the PLLA electrospun patch, elemental analysis of Pue, PLLA, and PLLA@Pue was performed by Fourier transform infrared (FT‐IR) spectroscopy. As shown in Figure 2e, the curves and peaks of PLLA and PLLA@Pue were basically the same. Compared to PLLA, a new peak appeared at 1513 cm^−1^ in the spectrum PLLA@Pue, which was consistent with the peak of Pue. These results further confirmed that Pue had been successfully grafted onto the PLLA electrospun patch and did not harm the PLLA structure.

Drug release from the PLLA@Pue electrospun patch was detected by an UV spectrophotometer. The maximum absorption wavelength of Pue is 251 nm, and the standard curve of the Pue solution was y = 0.0649x + 0.038 (R^2^ = 0.999) (Figure S1, Supporting Information). As shown in Figure 2f, the release rate of Pue reached 50% over the first 3 days, and then the release rate subsequently slowed and remained stable for more than 20 days for a total release rate exceeding 80%. Compared to other drug‐loaded electrospun patches used for POP, such as PLA‐ascorbic acid electrospun patch^[^ ^14a^ ^]^ and PLLA‐bFGF electrospun patch,^[^ ^4^ ^]^ PLLA@Pue patch did not show obvious drug burst release and had more stable and long‐lasting release characteristics. This may be related to the fact that Pue is slightly soluble in water. These results showed that PLLA@Pue can release Pue rapidly in the early stage and then maintain long‐term sustained release. Recent studies have reported that the acute inflammatory response mainly occurs 3–5 days after implantation, which further leads to the erosion of the patch and the formation of scar tissue.^[^ ^24^ ^]^ Therefore, the PLLA@Pue electrospun patch can release Pue rapidly in the first 3 days to reduce inflammation and then continue to be stably released to promote tissue regeneration.

### 2.2 In Vitro Biocompatibility Test

**Figure 3:**
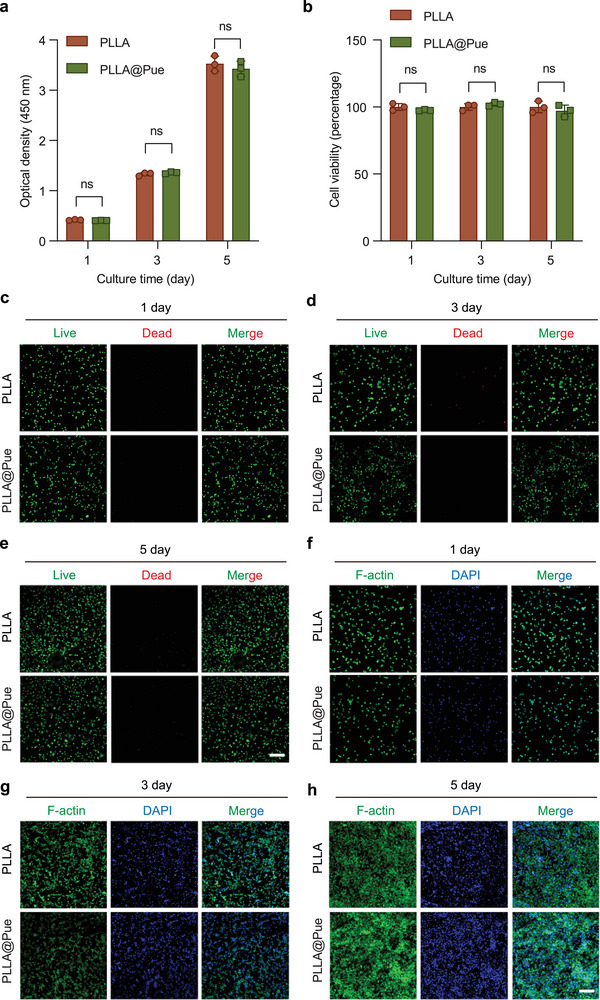
Biocompatibility test of PLLA and PLLA@Pue electrospun patches. a) CCK‐8 assays of L929 fibroblasts and b) cell viability on PLLA and PLLA@Pue electrospun patches after 1, 3, and 5 days of culture. c–e) Live/dead fluorescent staining (the red represented dead cells, while the green represented live cells) of L929 fibroblasts on PLLA and PLLA@Pue after 1, 3, and 5 days of culture. f–h) Fluorescence microscope of L929 fibroblasts on PLLA and PLLA@Pue after 1, 3, and 5 days of culture. Scale bar: 200 µm.

Good biocompatibility is a prerequisite for patches applied in POP therapy. The viability and proliferation of L929 fibroblasts were evaluated using a Cell Counting Kit‐8 (CCK‐8) assay. L929 fibroblasts were planted on PLLA and PLLA@Pue electrospun patches and cocultured for 1, 3, and 5 days. As shown in **Figure** **3**a, the OD_450_ values of each group increased gradually with the extension of culture time, and there was no significant difference between the two groups at each time point. Cell viability on both scaffolds exceeded 95% within the testing timeframe, which demonstrated the nontoxic nature of PLLA and PLLA@Pue. These results regarding cell proliferation and viability indicated that both PLLA and PLLA@Pue could provide an excellent microenvironment that mimics the ECM for cell growth and have good cytocompatibility.

Cytotoxicity was further evaluated by the live/dead assay, which assesses cell viability primarily based on cellular esterase activity and the integrity of the plasma membrane. In this assay, green fluorescence indicates living cells, and red fluorescence indicates dead cells. As shown in Figure 3c–e, each group mainly consisted of living cells, the amount of which increased significantly with the extension of culture time. Only a few dead cells were observed, and there were no significant differences between the two groups at each time point.

To visualize the cell morphology and distribution more intuitively, immunofluorescence staining of L929 fibroblasts cultured on the electrospun patches was performed with DAPI and Actin‐Tracker Green. The cell nuclei were stained with DAPI, and the cytoskeleton was stained with Actin‐Tracker Green. The cells were homogenously distributed on the electrospun fibers, and with the extension of culture time, the cells showed a clear increasing trend (Figure 3f–h). The cytoskeletons presented an irregular spindle shape, with oval nuclei located in the center. Notably, obvious pseudopodia connections between cells were observed on day 5, demonstrating excellent cell attachment, extension, and retention properties (Figure 3h). Collectively, these results showed that both the PLLA and PLLA@Pue electrospun patches has good biocompatibility with guaranteed safety for implantation in vivo.

### 2.3 In Vitro Anti‐Inflammatory and Pro‐Collagen Synthesis Assessments

It has already been proven that PLLA@Pue can release Pue in a sustained manner. In addition, Pue has been reported to effectively treat osteoarthritis by reducing the expression of inflammatory factors.^[^ ^19a^ ^]^ However, whether Pue has anti‐inflammatory and pro‐collagen effects in L929 fibroblasts in an inflammatory environment remains unknown. Therefore, the in vitro effects of Pue on L929 fibroblasts were investigated.

**Figure 4:**
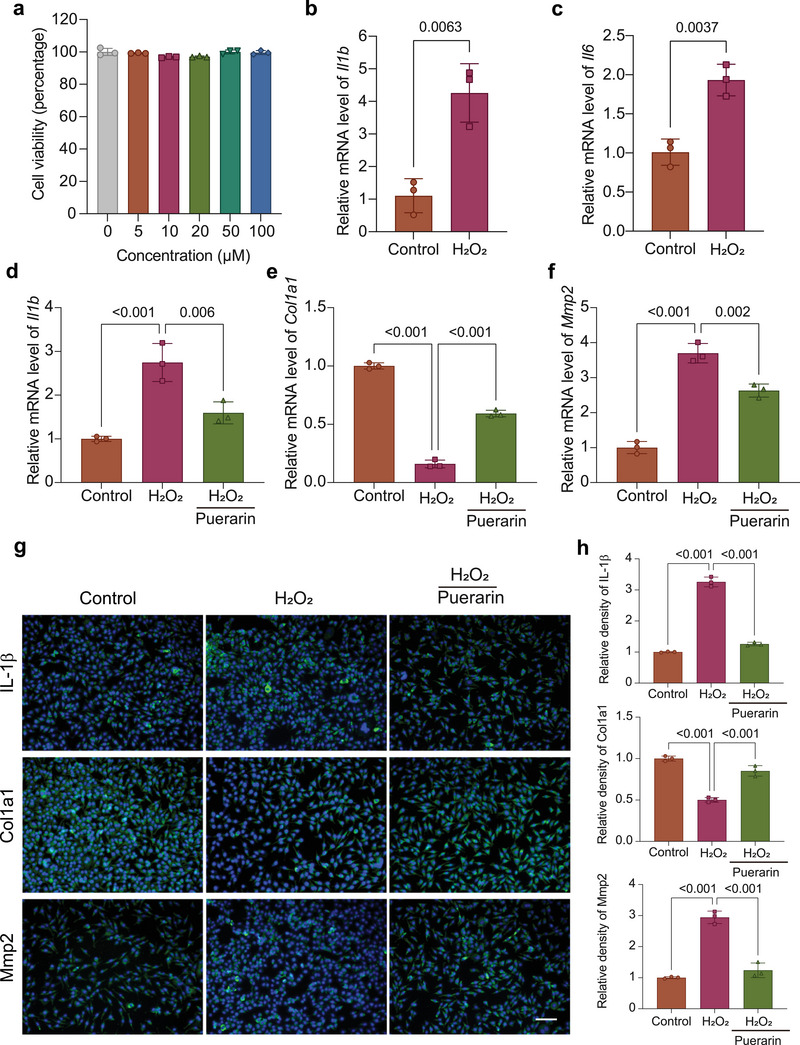
Effects of Pue on H_2_O_2_‐stimulated L929 fibroblasts. a) Viability of L929 fibroblasts treated with different concentrations of Pue after 1 day of culture. b and c) qRT‐PCR to test *Il1b* and *Il6* of L929 fibroblasts stimulated with or without H_2_O_2_. d–f) qRT‐PCR to test *Il1b*, *Mmp2* and *Col1a1* of H_2_O_2_‐stimulated L929 fibroblasts treated with Pue for 1 day. g) Immunohistochemical staining images of IL‐1β, Col1a1, and Mmp2, and h) their quantitative analysis of H_2_O_2_‐stimulated L929 fibroblasts treated with Pue for 1 day. Scale bar: 100 µm.

The viability of L929 fibroblasts treated with different concentrations of Pue was detected by a CCK‐8 kit. As shown in **Figure** **4**a, the cell viability exceeded 95% within the testing timeframe, indicating that Pue was biocompatible with L929 fibroblasts over a wide concentration range (5–100 µm). The viability of cells treated with 50 µm Pue was the highest, and so this concentration was used for the next test in this study.

To simulate an acute inflammatory response, L929 fibroblasts were treated with 500 µm H_2_O_2_ for 24 h according to previous literature reports.^[^ ^25^ ^]^ As shown in Figure 4b,c, the real‐time polymerase chain reaction (qRT‒PCR) results indicated that compared with the control group, the mRNA expression levels of inflammatory factors (*Il1b* and *Il6*) increased significantly after treatment with H_2_O_2_, indicating that the H_2_O_2_‐stimulated inflammatory L929 fibroblast model was successfully established. To investigate the anti‐inflammatory and pro‐collagen synthesis effects of Pue, H_2_O_2_‐stimulated L929 fibroblasts were treated with Pue (50 µm) for 24 h. As shown in Figure 4d–f, the qRT‒PCR results indicated that the mRNA expression levels of *Il1b* and *Mmp2* were significantly decreased after Pue treatment, while the mRNA expression level of *Col1a1* was significantly increased. This was consistent with the immunofluorescence staining results (Figure 4g,h). As one of the most common collagen‐degrading enzymes, Mmp2 is often upregulated and overactivated in the pelvic floor ligaments of POP patients. The results here confirmed that Pue could not only reduce the inflammatory response by suppressing the release of the proinflammatory cytokine IL‐1β but also promote collagen synthesis by downregulating the expression of Mmp2.

### 2.4 In Vivo Histological Analysis

An ideal patch for POP treatment should have the advantages of rapidly reducing inflammation after implantation in vivo, efficient integration into the surrounding tissue, and promoting the regeneration of healthy tissues.^[^ ^26^ ^]^ However, there are no mature animal models of POP currently, because it is difficult to simulate the comparable structural and functional changes in the pelvic floor which are caused by multiple risk factors such as childbirth.^[^ ^27^ ^]^ To demonstrate the practical value of PLLA@Pue, we established an abdominal wall muscle defect model in female rats to simulate the clinical manifestations of POP according to previously published protocols.^[^ ^4^, ^5^, ^8^, ^10^ ^]^ Histological analysis was used to evaluate the effects of tissue repair for the experimental groups of PLLA and PLLA@Pue electrospun patches and the control group without patch dressing treatment on days 3, 9, and 15.

**Figure 5:**
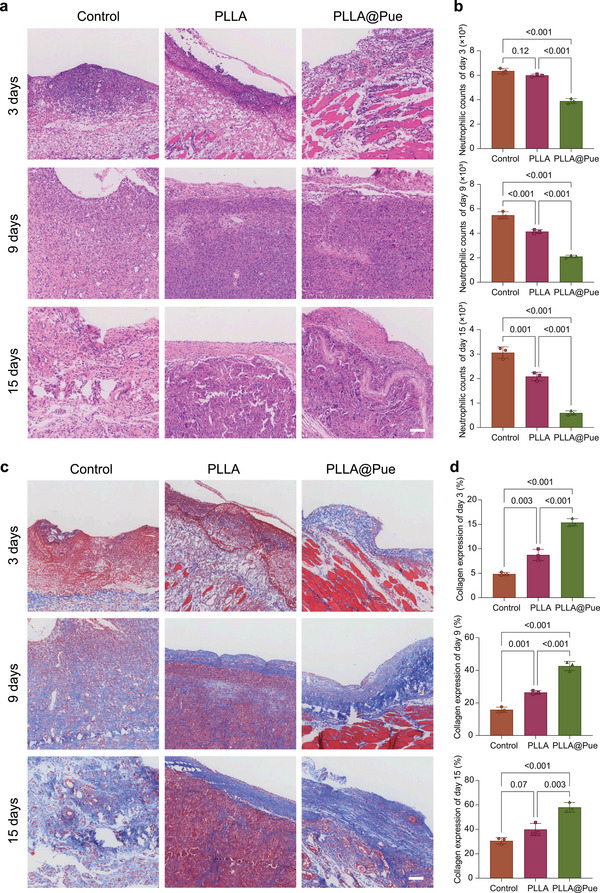
The observation of inflammation with HE detection and the observation of collagen regeneration with Masson staining. a) The HE images of abdominal wall muscle defect in PLLA and PLLA@Pue group 3, 9, and 15 days after surgery. b) Neutrophil count of PLLA and PLLA@Pue group 3, 9, and 15 days after surgery. c) The Masson images of abdominal wall muscle defect in PLLA and PLLA@Pue group 3, 9, and 15 days after surgery. d) Relative density of collagen deposited in the PLLA and PLLA@Pue group 3, 9, and 15 days after surgery. Scale bar: 100 µm. The data were presented as the mean ± SD from three different fields of microscope.

Inflammatory reactions and new tissue formation were investigated by hematoxylin and eosin (HE) staining. As shown in **Figure** **5**a,b, on day 3, acute inflammatory responses were observed in all three groups. Compared with the PLLA@Pue group, the control group and PLLA group showed a much more serious inflammatory response, more neutrophil infiltration, and looser connective tissue. On day 9, the inflammatory response began to subside in all three groups. The PLLA@Pue group had the lightest inflammatory response, and the number of neutrophils in the PLLA@Pue group also decreased most obviously. On the other hand, newly formed fibrous tissues were observed in all three groups. The regenerated tissue in the control group was the weakest. The regenerated tissue in the PLLA@Pue group was the thickest, and the boundary between the regenerated tissue and the surrounding normal tissue was not obvious, indicating the best healing effect. On day 15, the regenerated connective tissues in the PLLA@Pue group were mature and dense; a large number of fibroblasts were observed, while neutrophils were rare. On the contrary, in the control group, a large number of neutrophils still existed, while the newly formed tissue was weakly and loosely organized. In the PLLA group, the presence of moderate neutrophils was observed, indicating insufficient healing effect; However, the tissue repair speed in the PLLA group was faster than that of the control group.

Traditional commercial PP patch used in the clinic causes unfavorable acute inflammatory response, which often leads to implantation failure. The acute inflammatory response generally begins following neutrophil infiltration.^[^ ^28^ ^]^ Activated neutrophils synthesize chemokines, recruit macrophages, and ultimately affect the integration of the patch with the surrounding tissue.^[^ ^29^ ^]^ The above results suggested that the PLLA@Pue patch can effectively reduce the aggregation of neutrophils (on day 15, the mean neutrophil counts in the control group, the PLLA group, and the PLLA@Pue group were 3.059 × 10^3^, 2.086 × 10^3^, and 0.594 × 10^3^, respectively) and rapidly integrate into the surrounding tissue, thereby reducing the complications associated with the acute inflammatory response after mesh implantation in vivo.

To investigate the biological mechanism of the repair of abdominal wall defects, Masson's trichrome staining was carried out to evaluate the growth and deposition of collagen. In this assay, collagen fibers appear blue, and small muscle bundles appear red. The greater the density of the blue color, the denser the collagen deposition. As shown in Figure 5c,d, on day 3, there was no obvious blue coloration in the control group, and light blue fibers were sparsely distributed in the PLLA group and PLLA@Pue group. On days 9 and 15, the deposition of collagen gradually increased in all three groups. The PLLA@Pue group showed denser collagen deposition, the collagen bundles were arranged neatly and had no obvious boundary with the surrounding tissues. In contrast, the collagen fibers in the control group were disordered and had smaller blue‐stained areas. Compared with the PLLA@Pue group, the blue stained area of the PLLA group was smaller, indicating that the collagen deposition was insufficient; but the arrangement of collagen bundles was regular and tight compared with the control group, indicating that PLLA provided a good scaffold for collagen arrangement.

The weakening of pelvic connective tissues initiated by decreased collagen synthesis is a leading cause of POP. Therefore, continuous collagen production is a key factor in reducing the recurrence of POP after pelvic reconstruction surgery. The above results suggested that the PLLA@Pue patch could effectively promote the deposition of collagen (on day 15, the mean collagen expression in the control group, the PLLA group and the PLLA@Pue group were 30.55%, 39.84%. and 57.94%, respectively) and provide a good scaffold for collagen arrangement, thereby enhancing the mechanical properties of connective tissues and reducing the recurrence rate of POP in vivo.

### 2.5 In Vivo Inflammation and Collagen‐Related Factor Level Assessment

**Figure 6:**
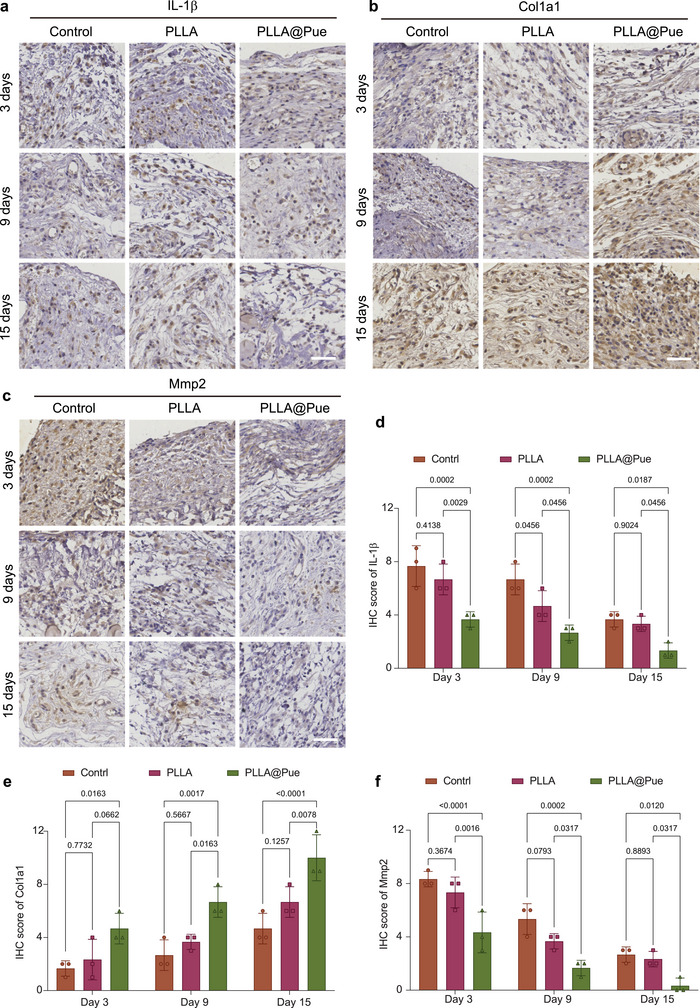
In vivo inflammation and collagen‐related factors. The IHC images of a) IL‐1β, b) Col1a1, and c) Mmp2 of abdominal‐wall muscle defect in PLLA and PLLA@Pue group 3, 9, and 15 days after surgery. The IHC scores of d) IL‐1β, e) Col1a1, and f) Mmp2 of abdominal wall muscle defect in PLLA and PLLA@Pue group 3, 9, and 15 days after surgery. Scale bars:50 µm. The data were presented as the mean ± SD from three different fields of microscope.

To investigate the mechanism of the PLLA@Pue electrospun patch in reducing inflammation and promoting collagen deposition in vivo, immunohistochemistry (IHC) staining was carried out to evaluate the changes in inflammation and collagen‐related factors. We obtained IHC staining images of IL‐1β, Col1a1, and Mmp2. IL‐1β has an inflammatory amplification effect, and evidences show that it plays a crucial role in the early stage of inflammatory response.^[^ ^30^ ^]^ An undesirable inflammatory response may lead to poor wound healing, resulting in immune rejection or even erosion of the patch. As shown in **Figure** **6**a,d, the expression of the proinflammatory factor IL‐1β was mainly concentrated around the tissue defects, and the IHC positive expression scores decreased with the extension of time in all three groups. On days 3, 9, and 15, the IHC scores of IL‐1β in the PLLA@Pue group were significantly lower than those in the control and PLLA groups, indicating that PLLA@Pue could effectively and continuously reduce inflammation by downregulating the expression of proinflammatory factor IL‐1β.

Col1a1 is an important gene encoding type I collagen, which mainly maintains the mechanical strength of the pelvic floor ligaments.^[^ ^31^ ^]^ IHC staining of Col1a1 was used to investigate collagen deposition more intuitively. As shown in Figure 6b,e, the expression of Col1a1 in all three groups showed an increasing trend over time. On days 3, 9, and 15, the IHC scores of Col1a1 in the PLLA@Pue group were significantly higher than those in the control group and PLLA group, indicating that PLLA@Pue could better promote collagen deposition and improve the mechanical strength of the new connective tissue.

Mmp2 is a key enzyme that regulates the degradation/remodeling of collagen. Overexpression of Mmp2 will accelerate collagen degradation and inhibit tissue repair.^[^ ^32^ ^]^ As shown in Figure 6c,f, the expression of Mmp2 in the PLLA@Pue group was significantly lower than those in the control group and PLLA group at all time points (days 3, 9, and 15), indicating that the PLLA@Pue may reduce collagen degradation and promote collagen synthesis by inhibiting the expression of Mmp2 in vivo. The above results provide reliable histological evidence for us to explore the anti‐inflammatory and pro‐collagen synthesis mechanism of the PLLA@Pue electrospun patch.

## 3 Conclusion

In this study, we successfully developed a drug‐loaded electrospun patch with anti‐inflammatory and pro‐collagen synthesis properties. The drug release experiment confirmed that Pue could be continuously released from the patch for more than 20 days. The introduction of Pue did not affect the mechanical properties or structure of the naked PLLA scaffold. In vitro biocompatibility tests indicated that the PLLA@Pue electrospun patches were nontoxic and promoted cell growth and proliferation. In vitro anti‐inflammatory and pro‐collagen experiments showed that Pue could reduce the inflammatory response by suppressing the release of IL‐1β and promote collagen synthesis by downregulating the expression of Mmp2. In vivo, experiments in rats showed that the PLLA@Pue electrospun patch could better promote the regeneration and repair of healthy tissues. Thus, we believe that this multifunctional patch can overcome the limitations of traditional patches and has great application prospects in POP pelvic floor reconstruction.

## 4 Experimental Section

### Materials, Cell Lines, and Animals

Pue was purchased from APExBIO, USA. PLLA was purchased from Jinan Daigang Co. (Jinan, China). Dichloromethane (DCM), dimethylformamide (DMF), and phosphate‐buffered saline (PBS) were purchased from Guangzhou Chemical Company (Guangzhou, China). Fetal bovine serum (FBS) was purchased from Invigentech Company, USA. High‐glucose Dulbecco's modified Eagle's medium (DMEM) and penicillin–streptomycin double antibiotics were obtained from Solarbio Company (Beijing, China). Standard L929 fibroblasts were provided by the Procell Life Science & Technology Co., Ltd., Wuhan, China. Female Sprague–Dawley (SD) rats (12 weeks old, 180–220 g in weight) were provided by the Animal Center of Sun Yat‐sen University (Guangzhou, China).

### Preparation of the PLLA@Pue Electrospun Patch

Briefly, PLLA (200 mg) was dissolved in a mixed solution of DCM/DMF (8:2 w/w, 2 mL), and the mixture was stirred at room temperature until the PLLA had completely dissolved. Then, Pue powder (8 mg) was added to the PLLA solution and mixed until a homogeneous solution formed before fabrication. The spinning solution was delivered into a needle of a 10 mL syringe and electrospun according to the following parameters: 15 cm receiving distance, 15 kV of high voltage, and a 0.8 mL h^−1^ flow rate. The collected samples were dried in a vacuum at room temperature to evaporate the residual organic solvents.

### Field Emission Scanning Electron Microscopy (FE‐SEM) Characterization

The electrospun patch samples were dried in a vacuum drying cabinet and fixed on a table with conductive adhesive. After pretreatment with gold spray, the micromorphology of the PLLA and PLLA@Pue electrospun patches was characterized by FE‐SEM (Quanta 200, Netherlands) with an accelerating voltage of 3–15 kV. Fiber diameters (n = 50) randomly chosen from the FE‐SEM images were measured via FIJI ImageJ software (2.14.0).

### Water Contact Angle (WCA) Measurements of the Electrospun Patches

The electrospun patch samples were cut into strips (40 × 15 mm) and fixed on glass slides. One drop (10 µL) of water was applied to the surface of the fibers, and the WCAs of the samples were determined by a goniometer (DS‐100, Kruss, Germany) at room temperature (n = 3).

### Fourier Transform Infrared (FT‐IR) Spectroscopy of the Electrospun Patches

The FT‐IR spectra of the Pue, PLLA and PLLA@Pue samples were acquired by an FT‐IR spectrometer (TENSOR‐27, Bruker, Germany) and analyzed using Origin 2021 software.

### Tensile Tests of the Electrospun Patches

The electrospun patch samples were cut into dumbbell shapes with dimensions of 60 × 10 mm. The tensile test was carried out by a universal mechanical testing machine (WD‐5A, Guangzhou Experimental Instrument Factory, China) at room temperature using a 50 N sensor. The speed of stretch was 30 mm min^−1^, and the stress‒strain curves were drawn with the tensile strength and fracture displacement values.

### In Vitro Drug Release Properties from the PLLA@Pue Electrospun Patches

PLLA@Pue samples were cut into 15 mm diameter circles and weighed, and the concentration of Pue was calculated. Each piece of electrospun patch was immersed in PBS (pH = 7.4, 10 mL) and placed in a constant temperature shaker (37 °C ± 0.5 °C, 100 rpm). At the selected time intervals (1, 2, 4, 8, 12, 24, 48, 72, 112, 168, 240, 312, 408, and 480 h), a sample of PBS (1 mL) was collected, and the corresponding amount of fresh PBS was returned to the reaction vessel. The concentration of Pue released from the electrospun patches was measured by an UV spectrophotometer (Persee, China) at the maximum absorption wavelength. Three samples were used to construct the drug release curve.

### In Vitro Biocompatibility of the Electrospun Patches

PLLA and PLLA@Pue samples were cut into circles, sterilized by UV light, and placed at the bottom of a 24‐well plate. L929 fibroblasts were cultured in DMEM containing 10% FBS. The L929 fibroblasts were then seeded into the electrospun patches at a density of 1 × 10^5^ per well and placed in a carbon dioxide cell incubator (37 °C, 5% CO_2_). Cell viability and proliferation on the electrospun patches were evaluated by using Cell Counting Kit‐8 (CCK‐8, Sigma‒Aldrich, USA). The cytotoxicity of the electrospun patches was evaluated by a live/dead cell assay, which was conducted with a Calcein AM/PI Double Staining Kit (Beyotime, China). Cell morphology and distribution on the electrospun patches were observed by fluorescence imaging. DAPI (Beyotime, China) was applied to show the cell nuclei, while Actin‐Tracker Green (Beyotime, China) was applied to display the cytoskeleton. All fluorescence images were observed under an inverted fluorescence microscope (Olympus IX73, Japan). The above assays were performed according to the manufacturer's recommendations.

### In Vitro Anti‐Inflammatory and Pro‐Collagen Synthesis Experiments

To determine the optimal drug concentration of Pue, the viability of L929 fibroblasts was assessed using a CCK‐8 kit (Sigma‒Aldrich, USA). L929 fibroblasts at a density of 1 × 10^5^ mL^−1^ were seeded in a 96‐well plate containing 10% DMEM. The L929 fibroblasts were then treated with 0, 5, 10, 20, 50, and 100 µm Pue for 24 h, followed by the addition of fresh DMEM (100 µL) containing CCK‐8 reagent (10 µL) to each well, and the cells were incubated for 1 h in the dark (37 °C, 5% CO_2_). The absorbance was then measured at a wavelength of 450 nm with a microplate reader.

### H_2_O_2_‐Stimulated Inflammation in Fibroblasts and Treatment with Pue

L929 fibroblasts were treated with H_2_O_2_ to establish a model of acute inflammation according to previous literature. Cultured L929 fibroblasts at a density of 1 × 10^5^ mL^−1^ were treated with H_2_O_2_ (500 µm) for 24 h, and untreated cells served as the control group. After establishing the acute inflammatory model, the damaged L929 fibroblasts were treated with Pue (50 µm) for 24 h, and damaged L929 fibroblasts without any treatment were used as the control group. The gene expression levels of proinflammatory cytokines (*Il1b* and *Il6*) and collagen‐related factors (*Mmp2* and *Col1a1*) in each group were analyzed by real‐time polymerase chain reaction (qRT‒PCR). Total RNA was extracted using TRIzol reagent and reverse transcribed into cDNA using a PrimeScript RT reagent kit (TaKaRa Bio, Inc., Otsu, Japan). The RNA transcription and qRT‒PCRs were optimized according to a previously published protocol. The designed sequences of the primers used are listed in Table S1 (Supporting Information). Expression levels were normalized to *Gapdh* expression. The secretion of IL‐1β, Col1a1, and Mmp2 was detected by immunofluorescence. The samples were fixed with 4% paraformaldehyde for 15 min and permeabilized with 0.3% Triton X‐100. Primary antibodies against IL‐1β (ABclonal, A16288, 1:100), Col1a1 (ABclonal, A16699, 1:100), and Mmp2 (Abcam, ab86607, 1:100) were added for incubation at room temperature for 2 h, followed by incubation with the secondary antibody (Invitrogen, A‐11008, A‐11001, 1:500) for 2 h in the dark. After removing the secondary antibodies, a DAPI staining solution was added to label the nuclei, and the cells were incubated at room temperature for 10 min. Images were captured by an Olympus fluorescence microscope (BX53, Olympus, Japan).

### In Vivo Animal Experiment

According to previously published protocols,^[^ ^5^, ^8^ ^]^ an abdominal wall muscle defect model was established in female rats to simulate the clinical manifestations of POP (Figure S2, Supporting Information). All rats were housed under standard pathogen‐free conditions in an animal facility and treated with strict adherence to current national guidelines on animal welfare. All rats were anesthetized by intraperitoneal injection of pentobarbital (2 wt.%, 1.8 mL kg^−1^) before the experiments. After shaving the abdomens of the rats, a U‐shaped abdominal incision was made. The subcutaneous tissue and muscle layer were removed, and the peritoneal tissue was preserved. Then, a round full‐thickness abdominal wall muscle defect with a diameter of 1.0 cm was created at the end. Twenty‐seven female Sprague‒Dawley rats weighing 0.2 ± 0.02 kg and aged 12 weeks were randomly divided into three groups as follows: control group (without treatment), n = 9; PLLA group, n = 9; and PLLA@Pue group, n = 9. On the 3rd, 9th, and 15th days post‐surgery, three rats in each group were sacrificed, and defects and the surrounding tissues were removed for histological analysis.

### Histological Analysis

The specimens were fixed in 4% paraformaldehyde solution for 24 h and then dehydrated in a gradient of anhydrous ethanol (50%, 70%, 85%, and 95%). According to the standard protocol, the specimens were embedded in paraffin and sectioned into 4 µm thick sections for routine histological processing. After dewaxing pretreatment, the sections were stained with HE and Masson's trichrome and for IL‐1β, Col1a1, and Mmp2 IHC. Histological images were randomly selected and quantitatively analyzed by FIJI ImageJ software (2.14.0). The IHC‐positive expression scores were determined according to a previously published protocol.^[^ ^5a^ ^]^

### Statistical Analysis

All data were presented as means ± standard deviation (SD), and statistical analysis was performed using *t*‐test, one‐way or two‐way analysis of variance (ANOVA). All the data were processed using GraphPad Prism Software. A value of *p* < 0.05 was considered statistically significant. All the quantifications of images were analyzed by ImageJ software on high‐resolution images.

## Conflict of Interest

The authors declare no conflict of interest.

## Author Contributions

D.Z., D.X., and X.B.H. contributed equally to this work. H.M.W., H.W., L.J., and Z.P.L. conceived the idea and designed the experiment. D.Z., D.X., X.B.H., Y.Q.W., F.X.T, X.S.Q., and W.W.L. conducted experiments and data analysis; D.Z, D.X., and X.B.H. wrote the manuscript.

## Supporting information

Supporting Information

## Data Availability

The data that support the findings of this study are available from the corresponding author upon reasonable request.
