# Supplementary material for: Puerarin‐Loaded Electrospun Patches with Anti‐Inflammatory and Pro‐Collagen Synthesis Properties for Pelvic Floor Reconstruction
Source: Adv Sci (Weinh). 2024 Mar 21;11(21):2308590. doi: 10.1002/advs.202308590 (PMC11151014; doi:10.1002/advs.202308590)
Supplement: Supplementary file 1 — Supporting Information [file ADVS-11-2308590-s001.pdf]

ADVANCED SCIENCE
Open Access

Supporting Information

for *Adv. Sci.*, DOI 10.1002/advs.202308590

Puerarin-Loaded Electrospun Patches with Anti-Inflammatory and Pro-Collagen Synthesis Properties for Pelvic Floor Reconstruction

Di Zhang, Dong Xu, Xiaobo Huang, Yingqi Wei, Fuxin Tang, Xiusen Qin, Weiwen Liang, Zhongping Liang, Lin Jin*, Hui Wang* and Huaiming Wang**

Supporting Information

Puerarin-Loaded Electrospun Patches with Anti-Inflammatory and Pro-Collagen Synthesis Properties for Pelvic Floor Reconstruction

Di Zhang[1#], Dong Xu[1#], Xiaobo Huang[2#], Yingqi Wei[3], Fuxin Tang[1], Xiusen Qin[1], Weiwen Liang[1], Zhongping Liang[4], Lin Jin[5*], Hui Wang[1*], and Huaiming Wang[1*]*

[1] Department of General Surgery (Colorectal Surgery), Guangdong Provincial Key Laboratory of Colorectal and Pelvic Floor Diseases, Guangdong Institute of Gastroenterology, Biomedical Innovation Center, The Sixth Affiliated Hospital, Sun Yat-sen University, Guangzhou 510655, China

[2] Department of Ophthalmology, Biomedical Innovation Center, The Sixth Affiliated Hospital, Sun Yat-sen University, Guangzhou, 510655, China

[3] Translational Medical Center, The First Affiliated Hospital of Zhengzhou University, Zhengzhou 450052, China

[4] The Sixth Affiliated Hospital of Guangzhou Medical University, Qingyuan People's Hospital

[5] International Joint Research Laboratory for Biomedical Nanomaterials of Henan, Zhoukou Normal University, Zhoukou 466001, China

# These authors contributed equally.

* Corresponding authors:

Huaiming Wang, Email: wanghm7@mail.sysu.edu.cn.

Hui Wang, Email: wang89@mail.sysu.edu.cn.

Lin Jin, Email: jinlin_1982@126.com.

Zhongping Liang, Email: Liangzp2006@126.com

Keywords: Pelvic organ prolapse; Drug delivery; Anti-inflammatory; Pro-collagen synthesis; Electrospun patch.

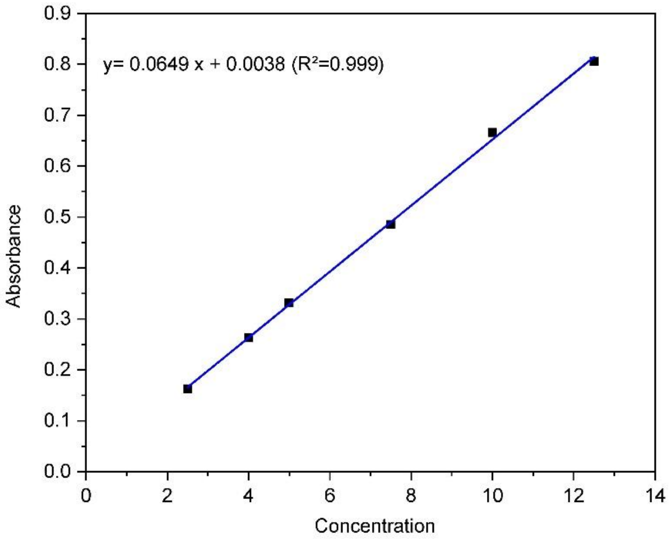

Figure S1. The absorbance - concentration standard curve of Pue. There is a good linear relationship between the absorbance and concentration of Pue solution in the range of 0.5-40 ug/ml.

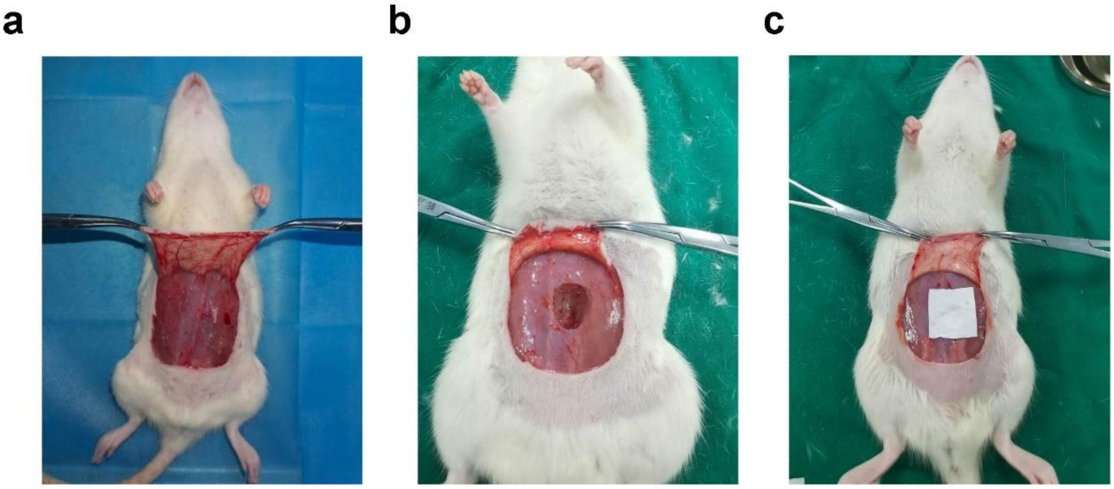

Figure S2. Photos of procedures for creating a full-thickness abdominal wall defect. a) The U-shaped abdominal incision of rat. b) The rounded full-thickness abdominal wall muscle defect with the diameter of 1.0 cm. c) The implants in vivo.

Table S1. The sequence of the primers used in the qRT-PCR amplification.

Genes	Forward primer sequences	Reverse primer sequences
Il1b	5’- CTGTGGCAGCTACCTGTGTC -3’	5’- CGTCACACACCAGCAGGTTA -3’
Il6	5’- TGCCTTCTTGGGACTGATGC -3’	5’- GCCTCCGACTTGTGAAGTGG -3’
Mmp2	5’- CCTGACCTGGACCCTGAAAC -3’	5’- CATGCTCCCAGCGTCCAAAG -3 ’
Col1a1	5’-CAGTGGTGAACCTGGTGCTC -3’	5’-CGGGCTCCTCGTTTTCCTTC -3’
Gapdh	5’- ATCATCTCCGCCCCTTCTGC -3’	5’- GAGCCCTTCCACAATGCCAA -3’
